# The Relationship Between Safety Climate and Performance in Intensive Care Units: The Mediating Role of Managerial Safety Practices and Priority of Safety

**DOI:** 10.3389/fpubh.2019.00302

**Published:** 2019-10-23

**Authors:** Patrick Teuma Custo, Rebecca Teuma Custo, Sandra Buttigieg

**Affiliations:** ^1^Cardiac Intensive Care Unit, Mater Dei Hospital, Msida, Malta; ^2^Intensive Therapy Unit, Mater Dei Hospital, Msida, Malta; ^3^Department of Health Service Management, Faculty of Health Sciences, University of Malta, Msida, Malta; ^4^Clinical Performance Unit, Mater Dei Hospital, Msida, Malta

**Keywords:** patient safety, safety climate, intensive care unit, safety procedure suitability, safety information flow, managerial safety practices, safety priority, empirical study

## Abstract

Patient safety is defined as the absence of preventable harm to a patient during the delivery of healthcare. Evidence from several reports and research studies reflect the high incidence and subsequent high cost of patient harm in general and within intensive care units. Against this background, this study tests a theoretical framework addressing relationships among patient safety climate dimensions and their impact on safety performance. The dimensions refer to safety in terms of procedure suitability and information flow, managerial safety practices, and priority of safety. A retrospective cross-sectional analytical research study was conducted. The target population was recruited from the three intensive care units in the main tertiary level hospital in Malta. A sample of 215 healthcare professionals, who fit the eligibility criteria, participated in this research study, achieving a response rate of 82.7%. The “Survey on Patient Safety Climate” was utilized. Findings support the following hypotheses: the higher the extent to which safety procedures are perceived as suitable to the intensive care units' daily work demands and processes, the lower the intensive care units' clinical incidents (*r* = −0.269, *p* ≤ 0.01) and the higher the extent to which safety information flow is perceived as clear and unambiguous to the intensive care units' daily work demands and processes, the lower the intensive care units' clinical incidents (*r* = −0.295, *p* ≤ 0.01). Findings also support the following hypotheses: managerial safety practices mediate the relationship between safety procedure suitability/safety information flow and clinical incidents (*p* = 0.009, *p* = 0.014, respectively) and priority of safety mediates the relationship between safety procedure suitability/safety information flow/managerial safety practices and clinical incidents (*p* = 0.002, *p* = 0.002, *p* = 0.042, respectively). Health service managers must ensure employees perceive safety procedures as suitable and safety information as clear and unambiguous, emphasize the manager's role as a safety referent and safety change agent and create an organization that prioritizes safety over work pace, workload and pressure for production. Essentially, health service managers need to create safety leaders to drive the organization to patient safety.

## Introduction

The World Health Organization (WHO) (1) defines patient safety as the absence of preventable harm to a patient during the delivery of healthcare. Categories of harm include falls and fractures, procedure related, therapeutic related, diagnostic related, drug related or operation related (2). Estimates show that in developed countries as many as one in ten patients is harmed while receiving hospital care (3). Also, ~40% of all healthcare spending is wasted due to poor quality care, resulting in additional hospitalization, litigation costs, infections acquired in hospitals, disability, lost productivity, and medical expenses. Subsequently, the WHO (3) recognizes patient safety as a serious global public health issue.

Concern over the levels of patient safety within hospitals was raised following a series of research studies, in the United States (US) (4–7) and in the United Kingdom (UK) (8). Events were single cases (for example: wrong site surgery, medication errors) or a number of patients had been killed either by the same errors committed by different professionals (for example: the Vincristine deaths) or by the same doctors making repeated errors (for example: the Manitoba and Bristol pediatric surgery fatalities) (9). Therefore, government agencies responded with a series of influential reports (9). In 1999, the US Institute of Medicine (IOM) and in 2000, the UK Department of Health published landmark reports *To Err is Human: Building a Safer Health System* (10) and *An Organization with a Memory* (11), respectively. More recently, *the Francis Inquiry Report* into the Mid-Staffordshire National Health Service Foundation Trust revealed widespread systemic failings among hospital staff (12, 13). The most alarming statistic is from the IOM report, which showed that between 44,000 and 98,000 people die in US hospitals each year as a result of medical errors at a cost of $17–$29 billion (10).

Within Intensive Care Units (ICUs), patient safety is also a problem. Rothschild et al. (14) conducted a prospective multidisciplinary epidemiologic study in the US: the Critical Care Safety Study. A total of 391 patients with 420 unit admissions were studied during 1,490 patient days. Findings indicated a high incidence of adverse events, preventable adverse events and serious errors (80.5, 36.2, 149.7 per 1,000 patient days, respectively). Garrouste-Orgeas et al. (15) conducted a prospective observational multi-center cohort study in France: the IATROREF study. A total of 70 ICUs and 1,369 patients were studied over a 1-week period. Findings also indicated a high incidence of medical errors. One thousand one hundred ninety-two medical errors were reported for 1,369 patients. The most common medical error was an error in insulin administration (185.9 per 1,000 days of insulin). Also, Kaushal et al. (16) conducted a prospective observational study in the US. Findings indicated that for 56 medical ICU patients the cost of an adverse event was $3,961. This extrapolated to annual costs of $853,000.

Therefore, this study aimed at developing a better understanding of the relationships among patient safety climate dimensions and their impact on safety performance. This is important to gain better insight on how to manage in non-routine work environments (17) and to shed light on the fact that managers need to move beyond formal aspects to ensure safety (18). Subsequently, this study examines the significance that employees perceive safety procedures as suitable, safety information flow as clear and unambiguous, managerial practices as emphasizing safety and safety is prioritized over work pace, workload and pressure for production. Also, this study aimed to add to the paucity of research on patient safety carried out in Malta and to provide groundwork for future research.

The main objectives are:

- To develop hypotheses based on theoretical models and findings from selected research studies from healthcare and industry,
- To gather data on healthcare professional's perceptions toward patient safety climate,
- To group the perceptions to reflect the four dimensions of safety climate: safety procedure suitability, safety information flow, managerial safety practices, and priority of safety,
- To test relationships between such dimensions based on the proposed hypotheses,
- To compare the findings to similar research studies carried out internationally and,
- Based on the findings, to identify recommendations for healthcare service management, and future research.

The research questions are:

- To what extent does safety in terms of procedure suitability and information flow predict the occurrence of the ICUs' clinical incidents?
- To what extent and how do managerial safety practices influence the relationships between safety in terms of procedure suitability/information flow and the ICUs' clinical incidents?
- To what extent and how does priority of safety influence the relationships between safety in terms of procedure suitability/information flow/managerial safety practices and the ICUs' clinical incidents?

## Methods

### Hypotheses Development

Throughout the years, a plethora of researchers have addressed safety culture, and/or safety climate. Following review, it was evident that at times the terms were used inadvertently interchangeably. Subsequently, for the purposes of this study, the terms were distinctively distinguished.

On the one hand, the term safety culture was first used in the aftermath of the Chernobyl nuclear disaster (19). The accident investigation report by the International Atomic Energy Agency described the accident arising through a poor safety culture at the plant and within the wider Soviet society (20). Therefore, safety culture was defined as “the product of individual and group values, attitudes, perceptions, competencies and patterns of behavior that determine the commitment to, and the style and proficiency of an organization's health and safety management” [(21), p. 23]. Throughout the years, various researchers have defined safety culture (in industry and healthcare) and examples are listed in Appendix 1.

On the other hand, the term safety climate first appeared in an investigation of safety attitudes in Israeli manufacturing by Zohar (22). Safety climate was defined as “a summary of molar perceptions that employees share about their work environments” [(22), p. 96]. Another popular definition of safety climate is that by Schneider (23). Safety climate was defined as “shared perception regarding the events, practices, and procedures, as well as the kind of behaviors that get rewarded, supported, and expected in a particular organizational setting” [(23), p. 384]. Again, throughout the years various researchers have defined safety climate (in industry and healthcare) and examples are listed in Appendix 2. Nevertheless, the distinction between culture and climate remains a source of debate and confusion in the safety field (24). Given that organizations are inherently hierarchical in structure, Flin (9) identifies multiple levels at which safety climate can be investigated. Hofmann and Stetzer (25) suggested that such levels include: individual, work groups, departments, organizations, and environments. Furthermore, according to Zohar and Luria (26), safety climate can be described in terms of two parameters: strength of climate (weak to strong) referring to the consensus concerning climate perceptions and level of climate (low to high) referring to the relative priorities of focal facets signified by climate perceptions. Moreover, a number of researchers (27–29) claim safety climate is a unidimensional variable while others (30, 31) claim is a multidimensional variable.

**Figure 1:**
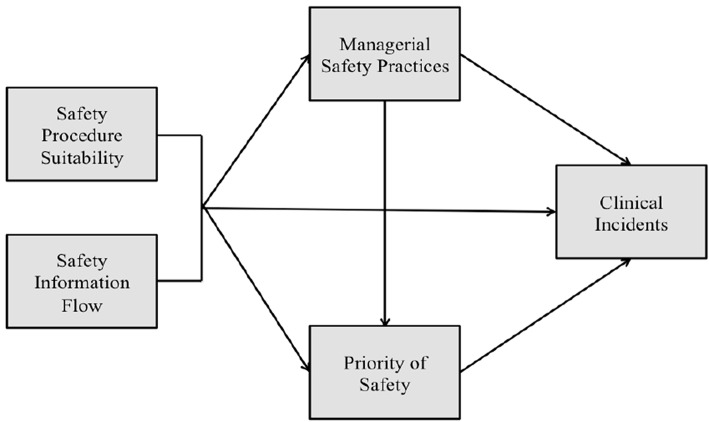
Proposed theoretical framework.

In industry, Griffin and Neal (32) produced and tested one of the first theoretical frameworks illustrating how safety climate relates to safety performance. In their model, the influence of safety climate on safety performance is mediated by worker knowledge, skill, and motivation. Furthermore, Neal and Griffin (33) explained that safety climate influences worker's knowledge and motivation, which in turn impacts on their safety behaviors and ultimately on safety outcomes. Christian et al. (34) built upon Neal and Griffin's (33) model of workplace safety. The researchers posited that situation related factors (safety climate and leadership) and person related factors (personality characteristics and job attitudes) are distally related to safety performance and even more distally related to safety outcomes. These factors are expected to impact more proximal person related factors such as safety motivation and safety knowledge that directly affect safety performance behaviors. Zohar (35) produced and tested a model where workers' behaviors-outcome expectancies mediate the relationship between their climate perceptions and safety behaviors. This is because expectations of how managers (and peers) respond to particular actions (for example: prioritizing safety over production targets) will to a significant extent determine which behaviors are executed. Flin (9) adapted the Griffin and Neal (32) and Zohar (35) models of workplace safety to show both patient and healthcare worker injuries as adverse outcomes. This study builds on the work conducted by Katz-Navon et al. (18) and Naveh et al. (17) and tests a theoretical framework (Figure 1) addressing relationships among patient safety climate dimensions and their impact on safety performance.

**a) Safety Procedure Suitability**
An organization is a complex system that develops a strategy to convert inputs to outputs (36). This process depends on four components: clinical work, people, formal structures and processes, and informal structures and processes (36). Nadler and Tushman (37) developed a simple pragmatic approach to such dynamics, known as congruency theory. This theory suggests that when the four components are aligned (or congruent) internally and with the strategy, the organization can perform effectively and produce quality outcomes. In turn, lack of congruency leads to failure to achieve the organization's targets.
Findings from Katz-Navon et al. (18) and Naveh et al. (17) support the hypothesis that the higher the extent to which procedures are perceived as suitable to the unit's daily work demands and processes, the lower the unit's treatment errors. This is in agreement with findings from Hofmann and Mark (38) and Singer (39) who explored safety climate in terms of its direct effects in healthcare settings addressing specifically patient safety. Findings from Hofmann and Mark (38) support the hypothesis that safety climate (conceptualized as “job duties allow for safe performance,” in conjunction with other safety climate dimensions) was negatively associated to medication errors and urinary tract infections. Also, findings from Singer (39) support the hypothesis that safety climate (conceptualized as “features of the organization,” in conjunction with other safety climate dimensions) was negatively associated with selected Agency for Healthcare Research and Quality's Patient Safety Indicators. This is also in agreement with findings from Mearns et al. (40) who explored safety climate in terms of its direct effects in industry. Findings support the hypothesis that safety climate (conceptualized as “satisfaction with safety activities,” in conjunction with other safety climate dimensions) was negatively associated to employees experiencing accidents and official accident reports.

In line with congruency theory, the following hypothesis is proposed:

1a. The higher the extent to which safety procedures are perceived as suitable to the ICU's daily work demands and processes, the lower the ICU's clinical incidents.

**b) Safety Information Flow**
However, safety procedure suitability may never cover all possible contingencies in non-routine work (17). Therefore, researchers (17, 18, 41) maintain that the way in which safety procedures are perceived and interpreted by employees is also associated with reduced treatment errors. Katz-Navon et al. (18) hypothesized and tested a curvilinear relationship between safety information flow and treatment errors. However, findings did not support a curvilinear relationship but suggest the possibility of a linear relationship. Despite this, findings from Naveh et al. (17) indicated a positive effect for safety information flow. Therefore, the higher the safety information flow, the higher the number of treatment errors. The researchers did not explain this result. However, this may simply reflect the fact that the higher the level of safety, the more treatment errors are disclosed rather than covered up. On the other hand, findings from Singer (39) in healthcare settings addressing specifically patient safety, support the hypothesis that safety climate (conceptualized as “interpersonal dynamics among individuals,” in conjunction with other safety climate dimensions) was negatively associated with selected patient safety indicators.
In industry, findings from Pousette et al. (42) support the hypothesis that safety climate (conceptualized as “safety communication” in conjunction with other safety climate dimensions) predicted safety behavior in a longitudinal study. Additionally, findings from Hofmann and Stetzer (27) support the hypothesis that group processes and functioning (the interaction that takes place among employees for example, communication, and coordination) is negatively associated with the number of recordable accidents. Also, findings from Mearns et al. (40) support the hypothesis that safety climate (conceptualized as “communication about health and safety,” in conjunction with other safety climate dimensions) was negatively associated with employees experiencing accidents and official accident reports.

In line with congruency theory, the following hypothesis is proposed:

1b. The higher the extent to which safety information flow is perceived as clear and unambiguous to the ICU's daily work demands and processes, the lower the ICU's clinical incidents.

**c) Managerial Safety Practices**
“Learning would be exceedingly laborious not to mention hazardous if people had to rely solely on the effects of their own actions to inform them what to do. Fortunately, most human behavior is learned observationally through modeling: from observing others, one forms an idea of how new behaviors are performed and on later occasions this coded information serves as a guide for action” [(43), p. 22]. This approach is known as Social Learning Theory and suggests that people learn through observing others' behaviors, attitudes, and outcomes of those behaviors (43). Therefore, observational learning is governed by four processes; attention, retention, reproduction, and motivation (43).
Managerial safety practices refer to the extent to which employees perceive their manager's behavior toward safety, which lets employees understand the extent to which the manager is committed to safety (17, 18). Zohar (35) argues that the essential dimension of safety climate is in fact, management commitment to safety and this solely suffices as a measure of safety climate. Zohar (22) indicated that in factories having successful safety programs there was a strong management commitment to safety. In low accident companies top management was personally involved in safety activities on a routine basis, whereas such commitment was conspicuously absent in high accident companies. In agreement with this, Zohar and Luria (26) contend that the most significant information is derived from episodes or occurrences that reveal management safety practices. Such episodes or occurrences serve as climate indicators that reveal the priority of key facets, which may differ from formal declarations (26).

### Managerial Safety Practices as a Mediator Variable

Throughout the years, research studies have empirically explored safety climate (conceptualized as solely managerial safety practices or in conjunction with other safety climate dimensions) in terms of its mediating effects on relationships between various variables and safety related outcomes. However, six of the eight research studies identified, recruited participants from industry whereas two recruited participants from health care, one addressed occupational safety while the other addressed patient safety.

Findings from Zohar (44), McFadden et al. (45), Martinez-Córcoles et al. (46), and Clarke (47) support the hypothesis that safety climate (conceptualized as solely managerial safety practices or in conjunction with other safety climate dimensions) mediated the relationship between leadership style and safety related outcomes. Zohar (44) and Clarke (47) tested both a transformational and transactional leadership style. On other hand, in healthcare addressing specifically patient safety, McFadden et al. (45) simply tested a transformational leadership style. Similarly, Martinez-Córcoles et al. (46) tested a leadership style based on the Empowering Leadership Model. This model identifies five different behaviors empowered leaders must show: leading by example, participative decision making, coaching, informing, and showing concern or interacting with employees.

Despite this, leaders may be transformational in one aspect of the job (achieving high production levels) but passive in other areas (achieving safety standards) (48). Subsequently, transformational leaders are not necessarily safety leaders (48). This is in agreement with findings from Barling et al. (29). Findings support the hypothesis that safety climate (conceptualized as solely managerial safety practices) mediated the relationship between safety specific transformational leadership and occupational injuries. Also, safety climate, safety consciousness, and safety-related events mediated the relationship between safety specific transformational leadership, role overload, and occupational injuries.

Findings from Zacharatos et al. (49) support the hypothesis that safety climate (conceptualized as management values in conjunction with other safety climate dimensions) mediated the relationship between human resource management practices related to high performance work systems and safety performance. Moreover, findings also supported the hypothesis that safety climate and trust in management mediated the relationship between human resources management practices related to high performance work systems and safety incidents. Also, findings from Wallace et al. (50) support the hypothesis that safety climate (conceptualized as solely managerial safety practices) mediated the relationship between management employee relationships and rate of accidents. Additionally, findings also supported the hypothesis that safety climate mediated the relationship between organizational support and rate of accidents.

In line with Social Learning Theory, the following hypotheses are proposed:

2a. Management safety practices mediate the relationship between safety procedure suitability and the ICU's clinical incidents.2b. Management safety practices mediate the relationship between safety information flow and the ICU's clinical incidents.

**d) Safety Priority**
The expectancy theory explains how an employee's behavior is determined by beliefs in three areas: expectancy (the extent to which increased effort will lead to improved performance), instrumentality (the extent to which improved performance will lead to a specified outcome), and valence (the extent to which that outcome is valued by the individual) (51). Performance is a function of skill and motivation. Skill relates to abilities (innate and acquired through, for example, safety procedures, and/or safety information flow) while motivation comprises the effort expended by the employee and the knowledge of what is expected by others (51). Effort is determined by the value to be derived as a result of the effort and the strength of the link between effort and the rewards. Expectancy theory leads employees to ask questions, such as: If I exert more effort is the safety goal attainable? Will the safety goal be rewarded? How much do I value the reward I will receive? Are alternative goals likely to be rewarded more highly? (52).
Safety priority refers to the extent to which employees' perceive safety as a top priority within the organization (17, 18). In other words, safety priority refers to the extent to which employees prioritize safety against work pace, workload, and pressures for production. Employees may base such priorities on their perception of probable consequences of safe or unsafe behavior (17, 18).

### Safety Priority as a Mediator Variable

Throughout the years, research studies have empirically explored safety priority as a mediator that influences the relationship between various variables and safety related outcomes. Griffin and Neal (32) argue that the value that individuals personally place on safety is an individual motivational construct rather that an aspect of safety climate. In view of this, the author included research studies that explored the dimension safety motivation to reflect the dimension safety priority as a mediator that influences the relationship between various variables and safety related outcomes. However, five of the eight research studies identified, recruited participants from industry whereas three recruited from healthcare, one addressed occupational safety, and two addressed patient safety.

In healthcare addressing occupational safety, Neal et al. (28) and in industry, Griffin and Neal (32) hypothesized that safety motivation and safety knowledge would mediate the relationship between safety climate (conceptualized as management values, safety communication, safety training, and safety systems) and safety performance. The researchers' model incorporated two dimensions of safety performance: compliance and participation. Safety compliance refers to core safety behaviors such as employees' adherence to safety rules, regulations, and procedures. Safety participation refers to employees' voluntary engagement or extra effort for safety that goes beyond formal role prescriptions such as participating in a safety committee, helping others with safety matters, or attending a voluntary safety meeting. This differentiation is based on an organizational model of task performance and contextual performance as components of job performance (53). Therefore, compliance activities are generally mandated while participatory activities are generally voluntary. Findings from both research studies indicated that safety motivation and safety knowledge mediated this relationship.

In a meta-analysis, Christian et al. (34) hypothesized that safety climate would positively influence safety performance (through safety knowledge and motivation) and to negatively influence outcomes. Safety climate was conceptualized as management commitment, human resource management practices, supervisor support, internal group processes, boundary management, risk, and work pressure. Findings indicated that safety knowledge and safety motivation mediated the relationship between safety climate and safety performance. A strong safety climate should encourage safe action either through reward or through principles of social exchange (32, 54, 55). Also, a strong safety climate should enhance safety knowledge due to an environment where safety information is communicated formally through training and informally through on-the-job discussions (34). Furthermore, the researchers hypothesized that safety climate would be more strongly related to safety participation than safety compliance, due to the voluntary nature of participation, and the motivational desire of employees to reciprocate manager actions regarding safety (34).

Also, findings from Clarke (56) indicated that perceived safety climate (safety priority and managerial safety practices) mediated the relationship between dimensions of psychological climate (job, role, group, leader, and organizational attributes) and safety behavior. As previously mentioned, finding from McFadden et al. (45) and Clarke (47) supported the hypothesis that safety climate (conceptualized as safety priority and managerial safety practices) mediated the relationship between leadership style and safety related outcomes. Similarly, findings from Zohar (44) supported the hypothesis that priority of safety mediated the relationship between leadership style and safety climate (conceptualized as managerial safety practices). Additionally, findings from Hofmann and Stetzer (27) supported the hypothesis that the tendency for employees to approach one another regarding safety activities (safety priority) mediated the relationship between group processes and functioning (the interaction that takes place among employees for example, communication and co-ordination; information flow) and safety related behavior.

In healthcare addressing patient safety, Naveh et al. (17) contend that, in addition to specific guidance on how to assure safety, safety procedure suitability, and safety information flow send a message about priority of safety. Additionally, the researchers argue that this has a significant role influencing treatment errors (17). Findings support the hypothesis that priority of safety mediated the relationship between safety procedure suitability and treatment errors. Furthermore, that priority of safety mediated the relationship between safety information flow and treatment errors.

In line with expectancy theory, the following hypotheses are proposed:

3a. Priority of safety mediates the relationship between safety procedure suitability and the ICU's clinical incidents.3b. Priority of safety mediates the relationship between safety information flow and ICU's clinical incidents.

As previously discussed, Zohar (22) indicated that in factories having successful safety programs there was a strong management commitment to safety. In low accident companies, this commitment was also exhibited when safety matters were given high priority in company meetings and production scheduling based on the conviction that safety is integral part of production systems and accidents are faults in the system. In view of this, the author maintained that managerial safety practices would send a message about priority of safety and this, in turn, would have a significant role influencing treatment errors.

In line with expectancy theory, the following hypothesis is further proposed:

3c. Priority of safety mediates the relationship between managerial safety practices and ICU's clinical incidents.

### Participants and Procedures

This study is a retrospective cross-sectional descriptive and analytical survey. The target population was recruited from the three ICUs in the main tertiary level hospital in Malta: the Intensive Therapy Unit (ITU), the Cardiac Intensive Care Unit (CICU), and the Neonatal Pediatric Intensive Care (NPICU). Inclusion criteria included all full and part-time healthcare professionals (HCPs) working in the previously mentioned ICUs. HCPs include all staff directly involved in patient care and have been working for at least 1 month in the ICU prior to the investigation. HCPs included charge nurses/midwives, practice nurses/midwives, deputy charge nurses/midwives, senior staff nurses/midwives, staff nurses/midwives, consultants, specialist registrars, higher/basic specialist trainees, and physiotherapists. HCPs on maternity or emigration leave were also contacted. A consecutive sample of HCPs who fit the eligibility criteria were asked to participant. Given that this sample reflected the total population of HCPs working within Maltese ICUs, a sample size calculation was not required.

### Measures

Data were collected through a structured self-administered questionnaire. The “Survey on Patient Safety Climate” was adopted from Naveh et al. (17). Permission to use the tool was sought and granted from Naveh et al. (17). Given that all HCPs either read their undergraduate course in English or were required to be fluent in English to work in Malta, it was deemed unnecessary to translate the questionnaire to Maltese. It was assumed that the English language allowed a good expression of ideas and experiences and prevented language barriers (57). The questionnaire was divided into seven sections: safety information flow, safety procedure suitability, priority of safety, managerial safety practices, clinical incidents observed, background information, and additional comments.

The dimension, safety procedure suitability, was assessed with five items statements originally adapted from Brusson and Jacobsson (58) while safety information flow, was also assessed with five items statements originally adapted from Hofmann and Stetzer (27) and O'Reilly (59). On the other hand, managerial safety practices were assessed with eight items originally adapted from Hofmann and Stetzer (27) and Zohar (54) while priority of safety was assessed with seven items adapted from Zohar (54). Item statements pertaining to each dimension are listed in Table 1.

**Table 1:** Item statements pertaining to each dimension.

**SAFETY PROCEDURE SUITABILITY**
•Safety rules and regulations are suitable for the daily activities of the unit
•There are written safety rules and regulations
•The safety rules and regulations relate to all work-related issues
•The safety rules and regulations are detailed enough
•The safety rules and regulations are practical
**SAFETY INFORMATION FLOW**
•There is a routine process of updating safety rules and regulations
•Employees are informed about potential hazards
•There are safety-training programmes
•Information about safety is distributed regularly
•Safety rules and regulations are presented in a simple and understandable format
**MANAGERIAL SAFETY PRACTICES**
•My supervisor praises us whenever he sees a job done according to the safety rules
•My supervisor approaches team members during work to draw their attention to safety issues
•My supervisor's attention is drawn to a worker who has violated a safety rule
•My supervisor is committed to adherence to safety rules and procedures
•My supervisor considers safety performance when evaluating performance and in promotion considerations
•My supervisor gets annoyed with workers who ignore safety rules and regulations
•My supervisor ensures there are no hazards in the department that can be harmful to staff health
•My supervisor creates an atmosphere in which people can say whatever they think
**PRIORITY OF SAFETY**
•In order to get the work done, one must ignore some safety aspects
•Whenever pressure builds up, the preference is to do the job as fast as possible even if that means less safety
•Human resource shortages undermine safety standards
•Safety rules and procedures are ignored
•Safety rules and procedures are nothing more than a cover-up in case of lawsuits
•Ignoring safety is acceptable
•It doesn't matter how the work is done as long as there are no accidents


Also, HCPs reported observed clinical incidents over the past 12 months. Four groups of clinical incidents were presented. These included equipment related, such as equipment failure; clinical practice related, such as infection control incidents; pharmaceutical related, such as the administration of the incorrect drug; and administration related, such as delays in patient admission or discharge. The categories of clinical incidents were adopted from Welters et al. (60).

The “Survey on Patient Safety Climate” is a psychometric validated tool having been tested for, face validity, content validity, construct validity, and internal consistency. Naveh et al. (17) did not present evidence of face or content validity. However, the researchers did perform exploratory and confirmatory factor analysis supporting an acceptable degree of construct validity. On the other hand, the author achieved an acceptable degree of face validity through the pilot study. A charge nurse, a deputy charge nurse, a senior staff nurse, a midwife, a consultant, and a physiotherapist who fit the eligibility criteria were asked to complete the questionnaire. Questions and queries were raised and discussed. No modifications were made to the “Survey on Patient Safety Climate” (Sections A–D). However, with regards to Section E titled “Clinical Incidents Observed” the option “non-applicable” was added. Such a modification was deemed necessary as, for example, physiotherapists do not administer pharmaceutical treatment, and are not able to report the perceived pharmaceutical related treatment errors over the past 12 months. With regards to Section F: question 6, the options charge nurse/midwife, deputy charge nurse/midwife and practice nurse/midwife were grouped as charge/deputy charge or practice nurse/ midwife. Similarly, the options Postgraduate Diploma, Masters of Science and Doctor of Philosophy were grouped as postgraduate degrees. Such modifications were deemed necessary to ensure anonymity. The author also achieved an acceptable degree of content validity when a member of Patient Safety and Quality Improvement Team (expert on safety) expressed favorable feedback when asked to review the questionnaire. Also, Naveh et al. (17) and the author derived the coefficient alpha. The coefficient alphas (derived by the author) ranged from 0.847 to 0.867. Given that this is above 0.7, the instrument was deemed reliable as maintained by Nunnally (61).

Permission was sought and granted from the University of Malta Research Ethics Committee prior to data collection. Following ethical approval, permission from the Chief Executive Officer, Data Protection Officer, Director of Nursing and Midwifery Services, Chairman of the Anesthesia Department and the Intensive Care Unit Charge Nurses/Midwives was sought and granted. The participant letter clearly stated that completion and return of the questionnaire indicated willingness to participate and, subsequently a written informed consent was not provided.

### Data Analysis

The data collected was inputted into the Statistical Package for the Social Sciences (IBM, SPSS) Version 23. For the purpose of this analysis, the scale pertaining to priority of safety was reversed as recommended by Naveh et al. (17). Given that in Malta there are only three ICUs, it was not feasible to analyze data at the work group level. Subsequently, relationships were tested at the individual level only. This was deemed appropriate as all three ICUs provide Level 2 (high dependency) and/or Level 3 (intensive care) care as defined by the Intensive Care Society document Levels of Critical Care for Adult Patients (62). Also, as specified by Luria (63), in order to understand the role of work group level and organizational level practices promoting safety behavior, it is necessary to start with the individual psychological processes of employees.

**Figure 2:**
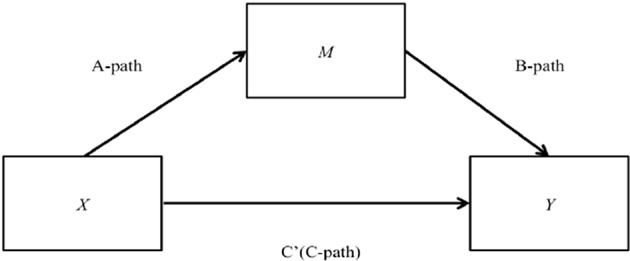
The mediation model.

**a)Correlation Analyses**
At the individual level, the relationships between safety procedure suitability and clinical incidents observed (1a) and safety information flow and clinical incidents observed (1b) were investigated using Pearson product-moment correlation coefficient (*r*). Standard multiple linear regression was performed to identify the best predictor (safety procedure suitability or safety information flow) of clinical incidents observed (64). The *F*-test was utilized to assess whether the set of independent variables collectively predicted the dependent variable while *R*-squared, the multiple correlation coefficient of determination, was utilized to determine how much variance in the dependent variable can be accounted for by the set of independent variables (65). The *t*-test was utilized to determine the significance of each predictor while standardized coefficients beta were used to determine the extent of prediction for each independent variable (65).
**b)Mediation Analyses**
At the individual level, the relationships between safety procedure suitability and clinical incidents observed mediated by managerial safety practices (2a) and safety information flow and clinical incidents observed mediated by managerial safety practices (2b) were tested. Also, at the individual level, the relationships between safety procedure suitability and clinical incidents observed mediated by priority of safety (3a), safety information flow and clinical incidents observed mediated by priority of safety (3b) and managerial safety practices and clinical incidents observed mediated by priority of safety (3c) were also tested. As illustrated in Figure 2, X is causing the mediator M and M is causing Y.
To test such hypotheses, while the Baron and Kenny (66) approach was consulted, Guillaume (67) maintains that the Baron and Kenny (66) approach does not quantify the indirect effect but infers mediation from a set of hypothesis tests and is prone to Type II error (not detecting an effect when there is one). In view of this, the PROCESS macro approach was conducted, Model 4 was utilized [Simple Mediation Model; (68)] and bootstrapping was employed (67).

A series of regression models were fitted:

- First, the mediator variable was predicted using the independent variables (A-path);
- Then, the dependent variable was predicted using both the independent variables (C') and the mediator (B-path);
- Finally, the dependent variable was predicted using the independent variables in the absence of the mediator variable (C-path). Therefore, the coefficient beta for A-path, B-path, C' and C-path, and the corresponding *p*-values were derived.

A measure for the indirect effect of X on Y was presented after the regression model. Therefore, the effect size was reported including limits of the 95% confidence intervals. Mediation occurred when the regression analysis of the independent variable on the dependent variable, controlling for the mediator, was statistically insignificant (67). On the other hand, mediation still occurred when the regression analysis of the independent variable on the dependent variable, controlling for the mediator, is still statistically significant. Some of the effect of the independent variable on the dependent variable would be through the mediator, but there would also be some direct effect (67). Subsequently, the coefficient beta for C' will be less than the coefficient beta for C-path. In view of this, the *p*-values of the coefficient beta for C' were reviewed. The Sobel's test was conducted to determine the significance of the indirect effect (69). Therefore, the z-values and the corresponding *p*-values were also derived.

## Results

### Response Rate, Demographic Data and Mean Scores Pertaining to Each Dimension

A response rate of 82.7% was achieved (215/260). 58% of respondents worked in ITU, 32% in NPICU and 10% in CICU. 66.1% were female while 33.9% were male. 70.2% were between 30 and 49 years old, 20.9% were between 18 and 29 years old while only 8.8% were older than 50 years. 44.2% had between 6 and 15 years of experience, 35.8% had between 1 and 5 years of experience, 10.2% had over 15 years of experience while 9.8% had more than 1 month experience but <1 year experience. 86% of respondents worked full-time while 11.6% worked reduced and 2.3% worked part-time. 55.3% of respondents were staff nurses/midwives, 12.1% were specialist registrars, 10.2% were senior staff nurses/midwives, 7.4% were higher specialist trainees, 6.5% were charge nurses/midwives, deputy charge nurses/midwives or practices nurses/midwives, 4.7% were consultants while 1.9% were senior physiotherapists and also 1.9% were basic specialist trainees. Lastly, 55.3% of respondents hold an undergraduate degree while 23.3% hold a postgraduate degree and 21.4% hold a diploma. The mean scores pertaining to each safety climate dimension were as follows: safety procedure suitability: 14.8 (possible range 5–25), safety information flow: 14.3 (possible range 5–25), managerial safety practices: 26.31 (possible range 8–40), and priority of safety: 24.78 (possible range 7–35).

### Correlation Analyses

Findings from correlation analyses (Table 2) indicated a small negative correlation between safety procedure suitability and clinical incidents observed (*r* = −0.269, *p* ≤ 0.01), with high levels of safety procedure suitability associated with lower levels of clinical incidents observed. Additionally, findings indicated a small negative correlation between safety information flow and clinical incidents observed (*r* = −0.295, *p* ≤ 0.01), with high levels of perceived safety information flow associated with lower levels of clinical incidents observed.

**Table 2:** The correlation matrix (**p ≤ 0.01, *p ≤ 0.05).

		**1**	**2**	**3**	**4**
1.	Safety information flow				
2.	Safety procedure suitability	0.694**			
3.	Managerial safety practices	0.519**	0.551**		
4.	Priority of safety	0.398**	0.475**	0.489**	
5.	Clinical incidents	−0.295**	−0.269**	−0.294**	−0.321**

Findings from standard multiple linear regression (Table 3) indicated that safety procedure suitability and safety information flow collectively predicted clinical incidents observed (*F*-test = 11.148, *p* ≤ 0.001). In the regression model, only safety information flow significantly predicted clinical incidents observed (safety procedure suitability *t* = −1.356, *p* = 0.177 vs. safety information flow *t* = −2.316, *p* ≤ 0.05).

**Table 3:** Findings from standard multiple linear regression (**p ≤ 0.001; *p ≤ 0.05).

	***F*-test**	***R*^2^**	**Safety procedure suitability**		**Safety information flow**	
			***t***	***b***	***t***	***b***
Clinical incidents	11.148**	0.095	−1.356	−0.123	−2.316*	−0.210*

### Mediation Analyses

Findings from mediation analyses (Table 4) indicated that managerial safety practices mediate the relationship between safety procedure suitability/safety information flow and clinical incidents observed (*p* = 0.009, 0.014, respectively). Also, findings indicated that priority of safety mediates the relationship between safety procedure suitability/safety information flow/managerial safety practice and clinical incidents observed (*p* = 0.002, 0.002, 0.042, respectively).

**Table 4:** Findings from mediation analyses.

	**A-path**	**B-path**	**C'**	**C-path**	**Indirect effect**	**CI (indirect effect)**	**Sobel (*z*)**	***p*-value**
1.	0.849	−0.1815	−0.205	−0.359	−0.154	−0.572 to −0.009	−2.590	0.009
2.	0.799	−0.167	−0.261	−0.394	−0.133	−0.277 to −0.005	−2.447	0.014
3.	0.656	−0.241	−0.207	−0.359	−0.159	−0.262 to −0.074	3.134	0.002
4.	0.550	−0.234	−0.266	−0.394	−0.129	−0.223 to −0.060	3.046	0.002
5.	0.439	−0.156	−0.156	−0.255	−0.099	−0.163 to −0.502	−0.793	0.042

*1. The indirect effect of safety procedure suitability on clinical incidents through the mediator, managerial safety practices*.

*2. The indirect effect of safety information flow on clinical incidents through the mediator, managerial safety practices*.

*3. The indirect effect of safety procedure suitability on clinical incidents through the mediator, priority of safety*.

*4. The indirect effect of safety information flow on clinical incidents through the mediator, priority of safety*.

*5. The indirect effect of managerial safety practices on clinical incidents observed through the priority of safety*.

**Figure 3:**
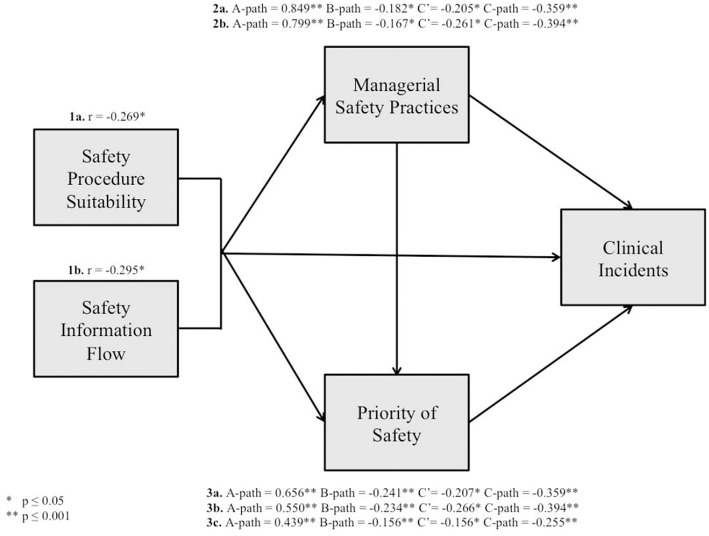
The tested theoretical framework.

Therefore, findings support the theoretical framework addressing relationships among patient safety climate dimensions and their impact on clinical incidents in Maltese ICUs. The tested theoretical framework is portrayed in Figure 3.

## Discussion

### Safety Procedure Suitability and Safety Information Flow

Within Maltese ICUs, findings indicated that to a moderate extent, employees perceived safety rules and regulations suitable for the daily activities of the unit, available as written policies or procedures, relevant to the work, detailed enough, and practical (17). Also, to a moderate extent employees perceived safety rules and regulations are updated regularly and presented in a simple and understandable format. Additionally, to a moderate extent employees felt that they are informed about potential hazards, safety-training programs are available while information about safety is distributed regularly (17).

In agreement with previously mentioned research studies, findings indicated a small negative correlation between safety procedure suitability/safety information flow and clinical incidents observed (*r* = −0.269, *p* ≤ 0.01; *r* = −0.295, *p* ≤ 0.01, respectively), with high levels of safety procedure suitability/safety information flow associated with lower levels of clinical incidents observed. According to Goodwin and Leech (70), a number of factors may have possibly affected the size of the correlation. Factors include: the amount of variability in either variable, differences in the shapes of the two distributions, lack of linearity in the relationship, the presence of one or more “outliers” in the dataset, characteristics of the sample used and measurement error. Despite this, it is important to note that the correlations were strongly statistically significant.

Findings from standard multiple linear regression, indicated that safety procedure suitability, and safety information flow collectively predicted clinical incidents observed (*F*-test = 11.148, *p* ≤ 0.001). However, in the regression model, only safety information flow significantly predicted clinical incidents observed (safety procedure suitability *t* = −1.356, *p* = 0.177 vs. safety information flow *t* = −2.316, *p* ≤ 0.05). These findings may possibly reflect the large correlation between safety procedure suitability and safety information flow (*r* = 0.694, *p* ≤ 0.01). Therefore, the variables are said to be collinear (71). Collinearity may cause problems in fitting and interpreting regression models, because the inclusion of two highly correlated variables in a regression model may give the impression that neither is associated with the outcome, even when each exposure is associated (individually) with the outcome (71).

Findings support congruency theory. Within an intensive care setting, findings from Pronovost et al. (72) indicated that the implementation of the central venous catheter (CVC) care bundle resulted in a large reduction of CVC infections (up to 66%) during the 18-months study period. However, findings from this study indicated that it is not simply the implementation of safety procedures that results in safety performance but it is the fact that employees deem safety procedures as suitable and subsequently comply with safety procedures. This is supported by findings from Resar et al. (73), which indicated that ICUs with the highest level of bundle compliance had the highest rate of infection reduction. Findings from this study also indicated that safety procedures must be accompanied by safety information flow, which is deemed clear and unambiguous. This is supported by evidence from Hawe et al. (74) who, designed and implemented a ventilator-associated pneumonia (VAP) prevention bundle in a medical and surgical ICU. The bundle was implemented, first passively then actively. Active implementation involved education, feedback of process and outcome measurements amongst other interventions. Subsequently, the active (rather than passive) implementation was associated with a significant improvement in VAP bundle compliance and decreased incidence of VAP.

### Managerial Safety Practices

Within Maltese ICUs, employees perceived that managers in their unit occasionally gave praise when s/he saw a job done according to safety rules, occasionally approached employees during work to draw attention to safety issues and occasionally approached employees who violate safety rules (17). Employees also perceived that managers in their unit occasionally committed to adhering to safety rules and procedures while occasionally considered safety when evaluating performance and/or in promotion considerations (17). Employees also perceived that managers in their unit occasionally got annoyed with workers who ignored safety rules and regulations and occasionally created an atmosphere in which people can say whatever they think (17).

In agreement with previously mentioned research studies, findings support the hypotheses that managerial safety practices mediate the relationship between safety procedure suitability/safety information flow and clinical incidents observed global (*p* = 0.009, *p* = 0.014, respectively). Findings support social learning theory. Such findings reflect the fact that when employees deem safety procedures as suitable and safety information flow as clear and unambiguous, managers receive messages that safety is important within the unit. This translates to managers' practices that emphasize safety. Therefore, the more employees perceive managerial safety practices as high, the more employees will learn about safety behavior (compliance and participation), and consequently less clinical incidents will occur. This is because managerial behavior provides cues regarding workplace norms and which kind of behavior is likely to be supported, valued or rewarded (75).

Interestingly, Zohar and Polachek (41) distinguish between sent and received role expectations and highlight the fact that this distinction is especially important when such expectations relate to priorities associated with competing role facets (41). Subsequently, when conflict arises between priority of safety and pressure for production, employees look at their managers for cues to guide their safety behavior (75). The assessment of priorities requires an interpretive sense making process on behalf of employees stemming largely from the difficulty of untangling and discriminating between espoused and enacted priorities (41). Espoused priorities refer to formal organizational safety policies by upper management while enacted priorities refer to the actual implementation and execution of safety procedures among employees (76). Therefore, employee perceptions essentially depend on received role expectations from enacted priorities rather than sent role expectation from espoused priorities (41). In addition to this, Zohar and Polachek (41) also emphasize the fact that changes in the content of received role expectations must remain stable and consistent offering sufficient opportunities for employees to experience and validate it as a real (rather than espoused) change (41). Changes in the content of received role expectations must be experienced in routine and daily leader-member exchanges rather than reserved to formal occasions, offering multiple opportunities for testing managerial enactment of espoused priorities (41). Consequently, a change of safety climate requires repetitive evidence indicative of sustained managerial prioritization of safe performance in the context of daily events or work situations presenting competing operational demands and it is this stability, which implicates genuine commitment to safety (41).

### Priority of Safety

Within Maltese ICUs, employees perceived that occasionally in order to get the work done, one must ignore some safety aspects, and whenever pressure builds up, the preference is to do the job as fast as possible even if it means less safety (17). Furthermore, employees perceived that occasionally human resource shortages undermine safety standards and occasionally safety rules and procedures are nothing more than a cover-up of lawsuits (17). Also, employees perceived that occasionally safety rules and procedures are ignored and occasionally ignoring safety is acceptable (17). In addition to this, employees perceived that occasionally it does not matter how the work is done as long as there are no accidents (17).

In agreement with previously mentioned research studies, findings support the hypotheses that priority of safety mediates the relationship between safety procedure suitability/safety information flow/managerial safety practices and clinical incidents observed (*p* = 0.002, *p* = 0.002, *p* = 0.042, respectively). Findings support expectancy theory. Findings reflect the fact that safety procedure suitability/safety information flow/managerial safety practices influence the priority of safety within the organization. Therefore, according to Naveh et al. (17), when safety procedures are deemed unsuitable employees deem priority of safety low within the unit. This is because complying with such procedures requires extra time and effort at the expense of speed and productivity. Subsequently, this situation calls for a trade-off between safety and productivity. Furthermore, according to Naveh et al. (17) an organization, which invests in safety training sessions and safety information distribution, sends the message to employees that not only productivity is important but that safety is also a central issue. Subsequently, employees should invest time and effort to maintain safety. Also, according to Naveh et al. (17), managers set the tone and tempo for the priority of safety by emphasizing specific safety behaviors and undermining others, by enforcing safety and by recognizing employee safety behavior.

### Under What Circumstances Are Managerial Safety Practices and Priority of Safety Critical for Safety Performance?

It is not only important to understand why managerial safety practices and priority of safety are critical for safety performance, but also under what circumstances are they critical for safety performance. Findings from Katz Navon et al. (18) and Bosak et al. (75) indicated that when priority of safety was high the impact of managerial commitment to safety on treatment errors or employees' risk behavior was nullified. Findings from Katz Navon et al. (18) indicated that employees received enough cues regarding the importance of safety within their unit via their understanding of priority of safety. However, Bosak et al. (75) found this effect only for conditions where employees experienced low levels of pressure for production. However, under conditions where employees experienced high levels of pressure for production, management commitment to safety was influential regardless of high vs. low priority of safety. Therefore, when conflict exists between pressure for production and priority of safety employees look at their manager for cues to guide their safety behavior. As managers have a direct bearing on the jobs and allocated rewards of employees, the likelihood of employees to engage in risk behavior is reduced when their manager is highly committed to safety despite a high demand for work place productivity.

Interestingly, findings from Hofmann and Stetzer (25) supported the hypothesis that role overload (pressure for production) was positively associated with the tendency to engage in unsafe acts (for example, adopting “short-cuts”). Also, findings from Hofmann and Mark (38) supported the hypothesis that patient complexity (pressure for production) moderated the relationship between safety climate (conceptualized as job duties allow for safe performance, social standing, management's attitude toward safety) and nurse back injuries and medication errors. In fact, *the Francis Inquiry Report* into the Mid-Staffordshire National Health Service Foundation Trust highlights the impact of staffing cuts on patient care (13). Inadequate staffing levels and staff workload (increased pressure for production) have been identified as key variables determining outcomes such as hospital mortality rates (77) and prolonged length of stay (78).

### Strengths and Limitations

This study adds value to patient safety literature. First, because the author did not simply measure the level of safety climate but approached the topic analytically. Second, because the author reviewed literature from various industries (for example metal processing plants, the food and beverage industry, the shipping industry) so as to gain “valuable insight about how to begin the process of improving the safety of healthcare” as advised by the IOM report [(10), p. 159]. Third, findings are representative of Maltese ICUs because the sample included the total population of HCPs working within Maltese ICUs and achieved a high response rate. However, given that this study included ICUs from Malta only, the ability to extrapolate findings is limited. Other limitations must be given due consideration. First, a cross sectional design was adopted and therefore it is difficult to differentiate between cause and effect from simple association (79). Also, reverse causality cannot be ruled out (42, 80). Second, given that throughout the years different researchers have conceptualized safety climate differently (44), the theoretical framework hypothesized and tested may be more complex. Third, a test-retest reliability procedure was not conducted to establish the stability of the tool. This is important as findings are at risk of acquiescence response bias, mood bias, non-response bias, social desirability bias as well as recall bias (81).

### Recommendations

Based on findings, health service managers are recommended to ensure employees perceive safety procedures as suitable and safety information as clear and unambiguous. This may be achieved by, for example, regularly assessing employees perceptions, regularly updating safety procedures in line with up-to-date evidence, encouraging discourse about safety, and/or using technology to improve access to safety information (for example: creating an app to easily access protocols or introducing clinical information systems to facilitate reminders and/or alerts). It is advisable that the managers receive safety training, which emphasizes their role as a safety referent (82), moving beyond safety policy formulation to that of a safety change agent (75). Also, managers are recommended to ensure safety is prioritized over work pace, workload and pressures for production. This may be achieved through the creation of a psychological contract between managers and employees which reflects the fact that safety behavior, is a behavior that is supported, valued, and rewarded (83). Essentially, managers must create good safety leaders who speak of safety, act safely at work, focus on maintaining safety standards, engage others in safety initiatives and recognize individual who adhere to safety (84). Along these lines, good safety leaders must engage in continuous quality improvement such as plan-do-study-act cycles and risk management (85).

Given that the cross-sectional design of this study makes it difficult to conclude whether safety climate is a lagging or leading variable, a longitudinal design is needed to strengthen the ability to infer causality. Future research should also identify and investigate other safety climate dimensions such as pressure for production (75), teamwork (86), non-punitive response of error (87), safety training (44), safety consciousness (29), and safety-specific leadership (48). A qualitative design or even a mixed method approach, such as triangulation may be adopted in an attempt to measure safety culture rather than climate (24, 88, 89). Future research should extend this study to other healthcare settings and analyze data at the work group, rather than at the individual level. However, in order to aggregate data from the individual level, the work group must have a strong safety climate (homogeneity of safety climate perceptions) (9). Essentially, future research should test a multilevel model of safety climate (31). Future research should investigate safety performance expressed as objective reliable variables such as incidence of ICU-acquired blood stream infections, ICU-acquired pressure ulcers and VAP. Lastly, future research should develop valid and reliable tools measuring safety behavior in terms of patient safety.

## Conclusion

This study enabled a better understanding of the relationships among patient safety climate dimensions and their impact on safety performance. This is important to gain better insight on how to manage in non-routine work environments (17) and shed light on the fact that managers need to move beyond formal aspects to ensure safety (18). This study emphasized the significance that employees perceive safety procedures as suitable, safety information flow as clear and unambiguous, managerial practices as emphasizing safety and safety is prioritized over work pace, workload, and pressure for production. Conclusively, this study adds value to patient safety literature, providing groundwork for future research.

## Data Availability Statement

The datasets generated for this study are available on request to the corresponding author.

## Ethics Statement

Permission was sought and granted from the University of Malta Research Ethics Committee prior to data collection. The participant letter clearly stated that completion and return of the questionnaire indicated willingness to participate and subsequently a written informed consent was not provided.

## Author Contributions

PT and SB developed the hypotheses and proposed theoretical framework, adopted the Survey on patient safety climate, PT recruited participants, collected data and conducted analysis including correlation and mediation analysis. SB supervised the research. PT, RT, and SB co-wrote the manuscript. All authors approved final manuscript.

### Conflict of Interest

The authors declare that the research was conducted in the absence of any commercial or financial relationships that could be construed as a potential conflict of interest.
