# Supplementary material for: The Relationship Between Safety Climate and Performance in Intensive Care Units: The Mediating Role of Managerial Safety Practices and Priority of Safety
Source: Front Public Health. 2019 Oct 23;7:302. doi: 10.3389/fpubh.2019.00302 (PMC6820301; doi:10.3389/fpubh.2019.00302)
Supplement: Supplementary file 1 [file Table_1.DOCX]

Appendix 1: Various definitions of safety culture as adopted from Wilson (91).

| Definition of Safety Culture | Domain | Reference |
| --- | --- | --- |
| ‘The objective measurement of attitudes and perceptions towards occupational health and safety issues.’ | General | Coyle, Sleeman, & Adams (92) p247 |
| ‘The collection of beliefs, norms, attitudes, roles and practices one uses while going about daily activities, including management decisions in a broader context.’ | General | Toft & Reynolds (1994), as cited in Kumar & Simpson (93) p330 |
| ‘Culture is a more complex and enduring trait reflecting fundamental norms, values, and assumptions that to some extent reside in societal culture.’ | Healthcare | Goodman (94) p25 |
| ‘A set of norms, beliefs, attitudes and practices, regarding universal precautions, shared between people in a certain place at a certain time.’ | Healthcare | Lymer, Richt & Isaksson (95) p548 |
| ‘When staff within an organization have a constant and active awareness of the potential for things to go wrong. Both the staff and the organization are able to acknowledge mistakes, learn from them, and take action to put things right.’ | Healthcare | National Patient Safety Agency (96) p2 |
| ‘One in which safety is everyone’s concern and there is an acknowledgement that errors can and will occur.’ | Healthcare | Dennis (97) p51 |
| ‘The assembly of characteristics and attitudes in organizations and individuals which establish that, as an overriding priority, safety issues receive the attention warranted by their significance. Safety culture is attitudinal as well as structural, relates both to organizations and individuals, and concerns the requirements to match all safety issues with appropriate perceptions and actions.’ | Nuclear | International Nuclear Safety Advisory Group (1991), as cited in Sorensen (98) |
| ‘All forms of learning behaviours which ‘add up to an shared commitment to think safely, to behave safely and to believe and trust in the safety measures put in place by the organization.’ | Nuclear | Lee (1993), as cited in Harvey, Erdos, Bolam, Cox, Kennedy & Gregory (99) p19 |
| ‘A subset of organizational culture, where the beliefs and values refer specifically to matters of health and safety.’ | Railway | Clarke (100) p185) |
| ‘The shared and learned meaning, experiences and interpretations of work and safety – expressed partially symbolically – which guides peoples’ actions towards risk, accidents and prevention.’ | Manufacturing | Richter & Koch (101) p705 |
| ‘A temporal manifestation of culture, which is reflected in the shared perceptions of the organization at a discrete point in time.’ | Offshore oil | Cox & Cheyne (102) p114 |
