# Supplementary material for: The Relationship Between Safety Climate and Performance in Intensive Care Units: The Mediating Role of Managerial Safety Practices and Priority of Safety
Source: Front Public Health. 2019 Oct 23;7:302. doi: 10.3389/fpubh.2019.00302 (PMC6820301; doi:10.3389/fpubh.2019.00302)
Supplement: Supplementary file 2 [file Table_2.DOCX]

| Definition of Safety Climate | Domain | Reference |
| --- | --- | --- |
| ‘The term ‘climate’ best describes employee perceptions, beliefs, and attitudes.’ | Healthcare | Goodman (103), p25 |
| ‘A subset of organizational climate, defined primarily in terms of employees’ perceptions. Safety climate is the safety culture, such as it appears to and is assessed by specific healthcare workers.’ | Healthcare | Coyle et al. (1995), as cited in Lymer et al. (104), p548 |
| ‘Molar perceptions people have of their work setting.’ | Construction | Dedobbeleer & Beland (105), p97 |
| ‘A set of perceptions or beliefs held by an individual and/or group about a particular entity.’ | Manufacturing/  Produce | Brown & Holmes (106), p 455 |
| ‘Perceptions of management’s commitment to safety, employee ownership of safety related issues, stereotyping of safety conscious employees, adherence to safety rules and procedures, and the existence of proactive approaches to managing safety.’ | Manufacturing | Garavan &O’Brien (107), p 146) |

Appendix 2: Various definitions of safety climate as adopted from Wilson (91).
